# Advances in HD-EMG interfaces and spatial algorithms for upper limb prosthetic control

**DOI:** 10.3389/fnins.2025.1655257

**Published:** 2025-09-04

**Authors:** Debora Quadrelli, Michele Canepa, Dario Di Domenico, Nicolò Boccardo, Michela Chiappalone, Matteo Laffranchi

**Affiliations:** ^1^Rehab Technologies Lab, Italian Institute of Technology, Genoa, Italy; ^2^Dipartimento di Informatica, Bioingegneria, Robotica e Ingegneria dei Sistemi (DIBRIS), University of Genoa, Genoa, Italy; ^3^Open University Affiliated Research Centre at Italian Institute of Technology (ARC@IIT), Genoa, Italy; ^4^Dipartimento di Elettronica e Telecomunicazioni (DET), Polytechnic of Turin, Turin, Italy

**Keywords:** high-density EMG, EMG recording Interfaces, myoelectric control, spatial information, machine learning, deep learning

## Abstract

Upper limb amputation significantly affects daily functioning and quality of life. Although myoelectric prostheses offer a promising avenue for restoring motor capabilities, high rates of device abandonment underscore challenges in control performance and user integration. Recent advances in high-density electromyography (HD-EMG) and machine learning (ML) algorithms have shown potential to enhance prosthetic dexterity. HD-EMG interfaces capture richer spatial and temporal muscle activation data, while ML algorithms exploit this information to improve intention detection and motion control. This mini-review explores advancements in HD-EMG acquisition systems, including both interface designs and recording technologies, as well as ML algorithms leveraging spatial information. In addition to summarizing the current state of the art, we discuss the challenges and the opportunities of embedding these technologies in prosthetic systems, with the objective of facilitating the translation of laboratory research into clinical applications.

## 1 Introduction

Dexterous hand and upper limb movements, facilitated by the coordinated activation of multiple muscle groups, are fundamental to human interaction with the environment. Upper limb amputation disrupts these essential functions, leading to significant limitations in daily living, reducing independence, and complicating work tasks and social interactions (Cordella et al., 2016). In response, prosthetic devices have become vital tools in restoring motor function, aiming to replicate the biomechanical and functional capabilities of the natural limb. Over the past two decades, upper limb prostheses, particularly poly-articulated systems, have undergone substantial technological advancement, offering increased functionality and customization to meet diverse user needs. However, as these devices become increasingly sophisticated, there is a growing demand for intuitive and robust control strategies capable of matching their complexity and dexterity, thus reducing both mental and physical effort. Among the various control approaches explored (Marinelli et al., 2023; Esposito et al., 2021), electromyography (EMG)-based methods are the most widely studied and implemented thanks to their ability to directly reflect voluntary muscle activation and, consequently, user intention (Merletti and Farina, 2016). The most advanced commercial solutions, myoelectric prostheses, utilize EMG signals derived from residual muscle contractions to control prosthetic movements. Despite their potential, the overall abandonment rates of upper limb prostheses is of approximately 44%, with myoelectric devices accounting for 92% of these cases (Salminger et al., 2022). This high abandonment rate is primarily attributed to inadequate control mechanisms (Salminger et al., 2022; Smail et al., 2021), which fail to manage the prostheses' available degrees of freedom in a natural and intuitive manner. Indeed, most commercial prosthetic devices rely on two surface EMG (sEMG) electrodes only, confining the control to one joint at a time and reducing functionality during complex tasks encountered in everyday life (Marinelli et al., 2023; Roche et al., 2014). To overcome these constraints, the growing emergence of artificial intelligence has led to increased interest in machine learning (ML) algorithms, which offer promising strategies for managing the complexity of multi-degree-of-freedom control. However, these approaches necessitate richer data input, thus prompting the development of high-density electromyography (HD-EMG) interfaces. These interfaces are based on the increased number of surface electrodes, typically more than 16, densely arranged over a smaller portion of the body, in contrast to standard low-density EMG configurations using up to 8 channels. By employing closely spaced electrodes placed on the skin above the target muscles, these high-density interfaces enable the generation of two-dimensional maps that represent the synergistic activation patterns of muscles during contraction (Merletti and Muceli, 2019). This capability to produce bi-dimensional representations has paved the way to leverage not only the temporal characteristics of EMG signals but also their spatial features within control algorithms for gesture recognition.

**Figure 1:**
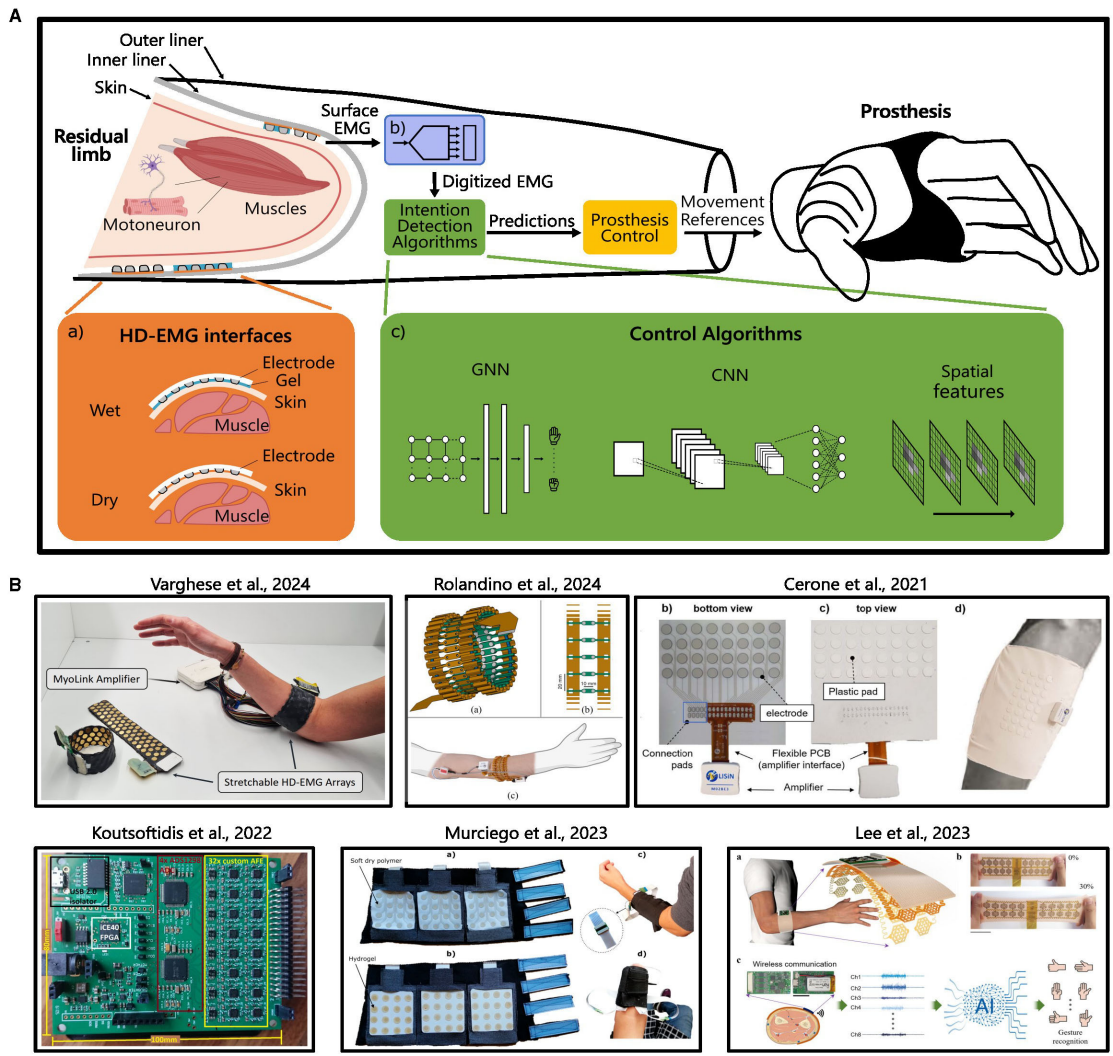
**(A)** Overview of HD-EMG interfaces components applied to the upper limb prosthetic control: electrodes are in contact with the residual limb skin, via a *wet* or *dry* interface (a). The socket is formed by an inner (stump interface) and outer rigid liner (structural hand interface): electrodes are located in the inner liner and the recording and control system are placed between the two liners. EMG signals are acquired via a multichannel analog-to-digital converter (ADC) and elaborated with a computing unit running the control algorithm (c) to decode user movement intention. Predictions are then mapped to prosthetic functions references for enabling joints actuation. **(B)** Overview of some reviewed article of HD-EMG interfaces and HD-EMG research-based recording systems.

Given the growing interest in HD-EMG interfaces and the emerging significance of spatial-based control strategies, particularly for embedded systems in upper limb prosthetics, this mini-review examines the current state of the art in these fields (Figure 1). The literature review was performed across major scientific databases such as Google Scholar, PubMed, IEEE Xplore, and Scopus. We used a combination of keywords including “High-Density EMG”, “EMG Recording Interfaces”, “EMG Recording”, “Myoelectric Control through Spatial Information”, “Graph Neural Network”, focusing on past decade's developments. While this work concentrates on acquisition systems and control strategies, comprehensive overviews of complete prosthetic devices can be found in existing reviews (Marinelli et al., 2023; Huang et al., 2024; Bates et al., 2020). By summarizing recent advancements and identifying key challenges in real-world implementation, this work aims to provide a foundation for future research and innovation in prosthetic applications.

## 2 HD-EMG acquisition systems

To the benefit of the discussion, we separate the HD-EMG acquisition system into two main subsystems: the *electrode interface* (Section 2.1) and the *recording system* (Section 2.2). These components are separately addressed in the following subsections.

### 2.1 HD-EMG interfaces

HD-EMG interfaces for upper limb prosthetics present significant challenges, with stringent technical and clinical requirements. Key technical factors include high electrode density for adequate spatial resolution, electrode diameters between 3 and 10 mm (Merletti and Farina, Merletti and Farina, 2016) , and proximal A/D conversion to minimize noise (Dumitru et al., 2025) . Clinically, critical requirements involve dry-contact interfaces, robust mechanical fixation for user comfort, and flexible, stretchable, full-circumference designs to accommodate various stump geometries. To date, no commercial prosthetic system exceeds 8 bipolar EMG channels. On the other hand, HD-EMG electrode interfaces, conceived for electrophysiology applications (Drost et al., 2006), offer significantly higher spatial resolution but are not yet suitable for practical prosthetic use. Conventional HD-EMG interfaces consist of arrays of monopolar electrodes arranged on a planar patch, having a conductive gel layer applied between the skin and the electrodes (i.e., wet electrodes, see Figure 1a) (Merletti and Cerone, 2020). The gel enhances signal quality by minimizing skin-electrode impedance and improves contact stability, thereby reducing movement artifacts. However, the need for gel represents a major barrier to usability and maintenance in everyday prosthetic applications. Other key aspects to consider are the electrode cabling arrangement, the electrodes encumbrance and the mechanical interface to the skin, including the material composition and the geometry. Ideally, the interface should conform to the anatomical variability of the residual limb and maintain stable contact during dynamic contractions. Moreover, ensuring consistent electrode placement across multiple donning and doffing cycles is crucial for minimizing data distribution shifts, which represent one of the most significant challenges in ML-based myocontrol prostheses (Kyranou et al., 2018). To address these limitations, several novel dry-electrode HD-EMG interfaces have been proposed, and although all following solutions are not ready for a clinical use, they were all validated in the lab.

These challenges led to the development of a flexible dry interface consisting of 32 copper electrodes mounted on interconnected stiffeners (Tam et al., 2019a). While effective, the use of multiple external wires limited its practicality. A subsequent design iteration (Tam et al., 2020) eliminated these cables, improving integration with prosthetic liners and enhancing wearability. Advancing toward even more flexible systems, a new solution based on textile (polyamide and elastene fabric) grid with 32 silver electrodes supported by polylactic acid (PLA) pads, and embedded wiring was developed (Cerone et al., 2021). Similarly, a 64 electrodes interface printed on a polyester and cotton textile substrate was introduced (Murciego et al., 2023). Alternatively, another highly flexible system relying on a thin polyethylene terephthalate (PET) sheet, with 64 screen-printed silver electrodes and integrated wiring was proposed (Moin et al., 2021). In addition to flexibility, recent efforts have explored stretchability as a key interface property. Although not high-density, a promising system was demonstrated effective for its stretchable Kirigami-based serpentine design (Lee et al., 2023). This hexagonal electrode pattern, supported by a breathable and adhesive substrate, enables excellent conformity to skin movements and promotes comfort by allowing vapor permeability. A more comprehensive solution combining flexibility, stretchability, and high sensor count was proposed through the development of an adjustable HD-EMG interfaces using elastic band combined with polyimide flexible printed circuit board (PCB) with 64 electrodes, which allow accommodation to various forearm sizes (Chamberland et al., 2023; Rolandino et al., 2024). Another novel flexible PCB-based solution covered with fabric and rubber layers was presented for its enhanced flexibility, stretchability, and wearability (Varghese et al., 2024). Despite these advantages, the connection cabling to the external MyoLink amplifier (Koutsoftidis et al., 2022) poses practical limitations for real-world application and remains a key barrier to clinical translation. A different solution proposed a semi-rigid system using a custom thermolyn liner embedded with 64 metal dome electrodes (Di Domenico et al., 2024). This system is designed to conform to the forearm and adapt to volume changes during muscle contractions, but it still relies on wired connections, which may reduce mechanical robustness and reliability.

An ideal EMG interface should incorporate high-density electrode count to enhance spatial resolution, while relying on dry electrodes to eliminate the need for conductive gel. However, eliminating the gel could compromise electrode-skin contact stability and signal quality. Additionally, for an ideal prosthetic integration, the interface must minimize inter-electrode cabling and be specifically designed for the incorporation within prosthetic sockets. A summary table is provided in the Supplementary material, detailing the design characteristics and key properties of the aforementioned EMG interfaces intended for embedded prosthetic applications. As shown in this comparative analysis, no single interface fully satisfies all requirements for clinical prosthetic integration. Despite significant advancements in the development of dry, flexible, stretchable, and minimally cabled systems, a critical limitation persists: the inadequate practical integration of these technologies within prosthetic sockets. Moreover, the absence of extensive validation under real-world conditions further underscores the gap between research and clinically viable solutions. Bridging this gap between laboratory prototypes and deployable clinical solutions remains a key objective for future research.

### 2.2 HD-EMG recording systems

Although some HD-EMG recording systems are commercially available, they do not fully meet the requirements for prosthetic integration. While most satisfy technical specifications, such as a sampling rate ≥2,000 Hz (Merletti et al., 2009; Farago et al., 2023) and ADC resolution of 12–24 bits (Merletti et al., 2009), the main limitation lies in embedding-related aspects. In particular, the acquisition system lacks a compact form factor suitable for integration into an embedded prosthesis near the electrode, which would eliminate the need for long analog signal paths (thereby reducing signal degradation), along with reliance on external signal centralization units. Below, we outline both commercial and research solutions, comparing their key attributes in Table 1. In this case as well, the proposed research solutions have been validated exclusively under laboratory conditions.

**Table 1:** Commercial and research-based HD-EMG recording systems with their key features for embedded prosthetic application.

	**General information**	**Mechanical**		**Sampling**		**Interface**	
	**Device**	**Dimensions [mm]**	Wearability*	**A/D res. [bits]**	**Data rate [Hz]**	**N. ch**	**Dry**
**Commercial**	Sessantaquattro+	73 × 105 × 12	✗	16	2,000	64	✓/✗
	MuoviPro (probe)	40 × 40 × 17	✗	16	2,000	32	✓/✗
	Trigno Maize Sensor	body: 50 × 30 × 13 head: 46 × 27 × 13	✗	n.d.	n.d	16	✓
	Trigno Avanti Sensor	27 × 37 × 13	✗	16	4,370	1	✓
	SAGA System (TMSi)	ø179 × 88	✗	24	500–4,000	32/64	✗
**Research**	Barone and Merletti (2013)	145 × 160 × 22	✗	24	2,441	24–64	✗
	Cerone et al. (2019)	34 × 30 × 15	✓	16	2,048	32	✗
	Cerone and Gazzoni (2017)	37 × 30 × 20	✓	16	2,048	32	✗
	Chamberland et al. (2023)	n.d.	✓	16	1,000	64	✓†
	Koutsoftidis et al. (2022)	100 × 80	✗	24	8,000	32	n.d.
	Lee et al. (2023)	n.d.	✓	n.d.	400	8	✓†
	Moin et al. (2021)	n.d.	✓	n.d.	1,000	64	✓†
	Pozzo et al. (2004)	140 × 95 × 62	✗	16	1,024/2,048	4 × 16	✗†
	Tam et al. (2019b,a)	20 × 65	✓	16	1,000	32	✓†
	Zhao et al. (2021)	105 × 110 × 90	✗	12/16/20	500–4,000	64–256	n.d.

*Wearability is evaluated in the context of prosthetic fitting, considering factors such as system dimensions, the distance between the recording system and electrodes, and the need for an additional external signal centralizer. †The system is coupled with custom interface.

Among commercial devices, the SAGA System (TMSi) supports up to 128 channels, offering high acquisition capacity. However, its large form factor significantly limits its applicability in embedded prosthetic settings. Similarly, the 64-channels portable wireless amplifier Sessantaquattro+ (2024) offers enhanced wearability, but remains too bulky for direct socket integration. A more compact option from the same manufacturer, is the MuoviPro (2023), which reduces physical encumbrance but still requires a separate wet reference electrode and an external signal centralization unit, a limitation for practical prosthetic use. A smaller alternative, with embedded dry reference electrode, is proposed by Delsys, with the 16-channels Trigno Maize Sensor (Delsys). However, its native skin interface, constrained in a 30 × 50 mm area, might limit forearm coverage for prosthetic application. These limitations highlight the need for co-designing electrode interfaces and recording units to meet the specific requirements of embedded prosthetic systems. A key enabler for such integration is the amplifier chip itself: in this regard, a valuable solution is provided by INTAN Recording with their RHD2000 series (INTAN Technologies LLC, 2012). The RHD chips are compact, low-power integrated circuits capable of amplifying, filtering, and digitizing electrophysiological signals up to 64 channels in a 9 × 9 mm package. This small, high-density configuration makes it particularly well-suited for the development of embedded solutions. In fact, different models of RHD2000 series chips have been employed in many research applications for recording systems (Cerone and Gazzoni, 2017; Cerone et al., 2019; Tam et al., 2019a,b; Chamberland et al., 2023).

As outlined in Table 1, most research-based systems achieve essential signal acquisition benchmarks, including high resolution and adequate sampling rates, while supporting a minimum of 32 channels. However, limitations persist in certain designs. Specifically, some systems are characterized by excessive bulk and a lack of integrated form factor, requiring long external cabling between the electrode array and recording unit (Pozzo et al., 2004; Barone and Merletti, 2013; Zhao et al., 2021). Such configurations not only compromise wearability but also introduce susceptibility to noise interference, thereby restricting their usability for prosthetic applications. Conversely, certain research-based systems surpass commercial solutions in terms of wearability by adopting a compact architecture, minimizing the physical distance between electrodes and acquisition circuit to preserve signal fidelity, while avoiding external signal centralizer.

In light of these considerations, future HD-EMG recording system for prosthetic applications should embody a miniaturized, wearable form factor; support high-resolution and appropriately sampled signal acquisition across the full EMG bandwidth; ensure close coupling between electrodes and acquisition electronics to mitigate noise; and incorporate low-power consumption and wireless data transmission capabilities to promote prosthetic applications.

## 3 Spatial-based control algorithms

Recently, advanced ML algorithms have emerged as promising alternative to traditional myoelectric control for upper-limb prostheses. In particular, growing interest has been directed toward algorithms that leverage spatial information (Figure 1c), driven by the development of HD-EMG interfaces. These methods provide high-resolution temporal and spatial activity information from the same data, expanding the range of neuromuscular characteristics that can be extracted, such as spatial distribution and muscular activation patterns, which are not accessible with conventional low-density EMG configuration.

For simplicity these algorithms are following categorized in spatial features-based, deep learning (DL) approaches, and Graph Neural Networks (GNNs). While the focus is on methods targeting motor execution of the prosthesis, real-time implementations remain limited in the literature. The studies presented primarily address intention detection, which, nevertheless, constitutes a critical first step toward full control.

Spatial features offer notable advantages for HD-EMG-based myoelectric control, primarily, they are more robust against signal variability. Unlike time-domain features, they are not solely dependent on signal amplitude, which makes them particularly effective in mitigating amplitude signal fluctuations caused by muscle fatigue, electrode displacement, or posture changes (Kyranou et al., 2018). Indeed, spatial features are demonstrated in several works to be particularly effective in combination with temporal, and spectral descriptors (Nougarou et al., 2019; Jaber et al., 2021a,b; Chen et al., 2023a; Ma et al., 2023). Additionally, some spatial features algorithms, were proved to support lower computational cost and time (Ma et al., 2023). Building on this concept, future researches could explore different EMG representation to leverage its spatial properties starting from images (Díaz-Amador et al., 2018), pharos (Onay and Mert, 2020), or 3D muscle activity reconstruction (Urbanek and Van der Smagt, 2016). However, spatial features alone lack temporal resolution, limiting their effectiveness in dynamic gesture recognition. Their performance also depends heavily on electrode grid density and placement, with suboptimal configurations reducing reliability. Thus, while spatial features strengthen robustness against signal instability and computational efficiency, their limitations must be addressed through careful system design and hybrid feature integration.

In parallel, DL approaches, and in particular Convolutional Neural Networks (CNNs), have gained interest due to their ability to automatically learn features, process large datasets, and extract spatial-temporal features (Chen et al., 2023b) while inherently offering a degree of translational invariance, an advantageous property for mitigating the effects of electrode displacement. Additionally, such methods are well-suited to be enhanced in combination with other architectures and pre-processing processes , such as array barrel shifting (Chamberland et al., 2023), data augmentation for proportional and simultaneous control (Sîmpetru et al., 2023), improved hand kinematics estimation (Sîmpetru, 2023), signal expansion and deformable convolution layers for performance optimization (Wang et al., 2022), and two-branches CNN architecture for more robust gesture recognition (Tam et al., 2024). However, these algorithms require large datasets, significant processing power and memory, which may limit their deployment in low-power or wearable systems.

More recently, Graph Neural Networks (GNNs) have been emerged as promising direction for upper limb prosthesis control, offering the possibility to model the spatial relationships between electrodes. By representing each electrode as a node and their physical or functional proximity as edges, GNNs can effectively capture topological relationships and dynamic muscle interactions that are not easily modeled using traditional algorithms. While widely studied in fields like social networks and molecular interactions, their application to EMG signals remains relatively unexplored. Pioneered work relied on graph-based HD-EMG representation to leverage the spatial information provided by sensors, by using 128 nodes (electrodes) and 884 edges (neighboring connections) (Massa et al., 2022). Despite promising results, excessive inter-node connections increased computational cost. To address this, a follow-up study (Massa et al., 2023) applied explainable AI to retain only essential edges, reducing complexity while maintaining performance. Unlike these electrode-structured graphs, a muscle connectivity-based graph, incorporating also a temporal module alongside spatial features, was also proposed (Zhong et al., 2023). Similarly, a spatio-temporal graph convolution module was presented (Xu et al., 2024). Recently, another spatio-temporal GNN with sensor-wise temporal connections, linking corresponding nodes across consecutive spatial graphs over successive time, was demonstrated (Lee et al., 2025). Although these GNN algorithms performed well in laboratory tests, their real-time applicability for prosthetic control remains untested. In practice, their deployment may be constrained by high computational demands, particularly with densely connected graphs and complex model architectures.

In conclusion, the spatial information encoded in HD-EMG constitutes a powerful means of enhancing myoelectric control. While a range of algorithms have been proposed to leverage this spatial richness, each presents distinct trade-offs, and comparative analysis, to define which approach outperforms the others, remains limited. Algorithm selection should therefore be application's needs-driven, balancing real-time performance, robustness, and computational constraints. In addition, in an ideal context, algorithm should run on energy-efficient, embedded platform capable of real-time processing and adaptation; however, current hardware often falls short of the demands of DL models, necessitating cloud-based or external computation, which introduces latency, bandwidth concerns, and security risks. As such, optimizing the trade-off between algorithmic complexity and hardware capability remains a key design constraint.

## 4 Integration of HD-EMG and spatial-based control algorithms

As observed, recent advancements in upper limb prosthetics focus on HD-EMG interfaces and sophisticated intention decoding algorithms that utilize spatial and positional information from electrode arrays. However, the next significant step in this field is integrating these components to fully maximize their potential.

Although a fully integrated system, comprising an HD-EMG interface, an HD-EMG recording system, and a computing unit with advanced algorithm, has not yet been realized in the current state of the art, some preliminary integration solutions have been proposed. For instance, Chamberland et al. (2023), as well as Tam et al. (2020) and Varghese et al. (2024), proposed a novel interface coupled with a CNN-based algorithm. However, in most studies presenting novel interfaces, control algorithms are typically employed for validation rather than as fully integrated components within embedded prosthetic systems. A notable integration effort, though not fully embedded, was introduced by (Moin et al., 2021), who presented an HD-EMG recording system with in-sensor adaptive ML for real-time prosthetic control. Conversely, when novel ML spatial-based algorithms are introduced, the primary focus is typically on the algorithm itself rather than on its integration with new interface designs. Indeed, most studies reviewed in Section 3 lack in pairing their algorithms with novel user interfaces. Instead, they utilized signals acquired from commercial wet (such as in Sîmpetru et al., 2023; Sîmpetru, 2023), or dry electrode solutions (such as in Xu et al., 2024), or relied on public dataset (as in Wang et al., 2022; Massa et al., 2023; Lee et al., 2025).

Regarding fully integrated prosthetic systems, (Nguyen, 2021) proposed a system incorporating a peripheral nerve signal recording device and a recurrent neural network. While this approach represents progress toward integrated prosthetics, the system remains bulky and impractical for real-world applications.

These recent advancements clearly indicate a promising new direction in upper-limb prosthetics, where high-resolution sensing and advanced algorithm are converging toward integration. However, they also highlight several critical challenges, in both hardware and software design, that must be addressed to exploit the full potential of such embedded systems. From the hardware perspective, key areas for future development include the design of dry HD-EMG interface with minimal cabling to enhance wearability, the miniaturization of acquisition electronics to enable compact and lightweight recording units positioned near the electrodes, and the integration of low-power, high-efficiency processing units between the prosthetic socket and the liner. Conversely, on the software side, the implementation of computationally efficient, real-time, and robust intention decoding algorithm remains a primary challenge. Algorithms must be optimized not only for improving accuracy and robustness to signal variability, such as electrode shift, muscle fatigue, and environmental noise, but also for execution on resource-constrained embedded platforms (Zanghieri et al., 2019). Addressing these limitations will be essential for the development of the next generation of embedded prosthetic systems.

## 5 Conclusions

The integration of HD-EMG interfaces and advanced ML algorithms represents a significant improvement in prosthetics, enabling more precise, intuitive, and multi-degrees control of upper-limb devices. HD-EMG interfaces provide more spatial and temporal data, while ML algorithms can extract insights from this data to improve gesture recognition and motion control. However, translating this potential into real-world solutions remains an ongoing challenge. Real-world implementation requires stable and high-quality signals, compact hardware, and robust real-time control. In addition, daily usability requires interfaces that ensure accurate fitting and easy wearability. Facing these hardware and software challenges will be central to the next stage of progress in prosthetic development, as it is crucial for enabling reliable, high-dexterity devices suitable for everyday use. Ultimately, moving from lab setups to wearable systems will support clinical validation in real-world conditions, improve user experience, and reduce prosthesis abandonment.
