# Supplementary material for: Advances in HD-EMG interfaces and spatial algorithms for upper limb prosthetic control
Source: Front Neurosci. 2025 Sep 4;19:1655257. doi: 10.3389/fnins.2025.1655257 (PMC12443821; doi:10.3389/fnins.2025.1655257)
Supplement: Supplementary file 1 [file Data_Sheet_1.pdf]

Supplementary Material

Table S1. HD-EMG interfaces with their key features for embedded prosthetic application.

	**Flex-ible**	**Stretch-able**	**Full-circum.**	**N. ch**	**Electrode**	**Dry**	**$\varnothing_{electr.}$ [mm]**	**In-sensor A/D**	**Prosthetic fitting**
Cerone et al. (2021)	✓	✓	✗	32	Intexar (silver) pads	✓	10	✓	✗
Chamberland et al. (2023)	✓	✓	✓	64	ENIG pads	✓	7	✓	✗
Di Domenico et al. (2024)	✗	✗	✓	64	Steel domes	✓	8.5	✗	✓
Lee et al. (2023)	✓	✓	✓	8	copper on polyimide-support	✓	hexagonal w:12.6, h:20.0	✓	✗
Moin et al. (2021)	✓	✗	✓	64	Silver ink pads	✓	4.3	✓	✗
Murciego et al. (2023)	✓	✗	✓	16	IDUN DRYODE ink pads	✓	6	✗	✗
Rolandino et al. (2024)	✓	✓	✗	64	Silver pads	✓	4	✓	✗
Tam et al. (2019)	✓	✗	✗	32	Copper PCB pads	✓	8	✓	✓
Tam et al. (2020)	✓	✗	✗	32	Gold-plated pads	✓	8	✓	✓
Varghese et al. (2024)	✓	✓	✓	64	ENIG pads	✓	10	✗	✗

REFERENCES

Cerone, G. L., Botter, A., Vieira, T., and Gazzoni, M. (2021). Design and characterization of a textile electrode system for the detection of high-density sEMG 29, 1110–1119. Publisher: IEEE

Chamberland, F., Buteau, , Tam, S., Campbell, E., Mortazavi, A., Scheme, E., et al. (2023). Novel wearable HD-EMG sensor with shift-robust gesture recognition using deep learning

Di Domenico, D., Boccardo, N., Marinelli, A., Canepa, M., Gruppioni, E., Laffranchi, M., et al. (2024). Long-term upper-limb prosthesis myocontrol via high-density sEMG and incremental learning Publisher: IEEE

Lee, H., Lee, S., Kim, J., Jung, H., Yoon, K. J., Gandla, S., et al. (2023). Stretchable array electromyography sensor with graph neural network for static and dynamic gestures recognition system 7, 20

Moin, A., Zhou, A., Rahimi, A., Menon, A., Benatti, S., Alexandrov, G., et al. (2021). A wearable biosensing system with in-sensor adaptive machine learning for hand gesture recognition 4, 54–63. Publisher: Nature Publishing Group UK London

Murciego, L. P., Komolafe, A., Peřinka, N., Nunes-Matos, H., Junker, K., Díez, A. G., et al. (2023). A novel screen-printed textile interface for high-density electromyography recording 23, 1113. Publisher: MDPI

Rolandino, G., Zangrandi, C., Vieira, T., Cerone, G. L., Andrews, B., and FitzGerald, J. J. (2024). HDE-array: Development and validation of a new dry electrode array design to acquire HD-sEMG for hand position estimation Publisher: IEEE

Tam, S., Bilodeau, G., Brown, J., Gagnon-Turcotte, G., Campeau-Lecours, A., and Gosselin, B. (2019). A wearable wireless armband sensor for high-density surface electromyography recording (IEEE), 6040–6044

Tam, S., Boukadoum, M., Campeau-Lecours, A., and Gosselin, B. (2020). Forearm high-density electromyography data visualization and classification with machine learning for hand prosthesis control (IEEE), 722–727

Varghese, R. J., Pizzi, M., Kundu, A., Grison, A., Burdet, E., and Farina, D. (2024). Design, fabrication and evaluation of a stretchable high-density electromyography array 24, 1810
